# Impact of surface charge density modulation on ion transport in heterogeneous nanochannels

**DOI:** 10.1038/s41598-024-69335-1

**Published:** 2024-08-08

**Authors:** Amin Alinezhad, Mahdi Khatibi, Seyed Nezameddin Ashrafizadeh

**Affiliations:** Research Lab for Advanced Separation Processes, Department of Chemical Engineering, Iran University of Science and Technology, NarmakTehran, 16846-13114 Iran

**Keywords:** PNP nanotransistor, Bipolar smart nanochannel, Depletion zone, Surface charge density, Electroosmotic flow, Analytical chemistry, Chemical engineering, Electrochemistry, Physical chemistry, Theoretical chemistry

## Abstract

The PNP nanotransistor, consisting of emitter, base, and collector regions, exhibits distinct behavior based on surface charge densities and various electrolyte concentrations. In this study, we investigated the impact of surface charge density on ion transport behavior within PNP nanotransistors at different electrolyte concentrations and applied voltages. We employed a finite-element method to obtain steady-state solutions for the Poisson–Nernst-Planck and Navier–Stokes equations. The ions form a depletion region, influencing the ionic current, and we analyze the influence of surface charge density on the depth of this depletion region. Our findings demonstrate that an increase in surface charge density results in a deeper depletion zone, leading to a reduction in ionic current. However, at very low electrolyte concentrations, an optimal surface charge density causes the ion current to reach its lowest value, subsequently increasing with further increments in surface charge density. As such, at ${V}_{app}=+1 \text{V}$ and ${C}_{0}=1 \text{mM}$, the ionic current increases by 25% when the surface charge density rises from 5 to 20 $\text{mC}.{\text{m}}^{-2}$, whereas at ${C}_{0}=10 \text{mM}$, the ionic current decreases by 65% with the same increase in surface charge density. This study provides valuable insights into the behavior of PNP nanotransistors and their potential applications in nanoelectronic devices.

## Introduction

The advancement of techniques in fabricating charged nanopores/nanochannels with diverse geometries has opened up new opportunities for various applications, such as energy conversion^1–8^, molecular sensing^9–12^, tuning of Taylor dispersion^13,14^, ionic nanodiodes^15–18^, ionic nanotransistors^19–21^ and sensors^22–24^. One key characteristic of nanochannels is their comparable size to the electric double layer (EDL), characterized by the Debye length (${\uplambda }_{D}$). Under normal conditions, ${\uplambda }_{D}$ typically ranges from 1 to 100 nm. When nanochannel dimensions are reduced to the nanoscale, ion transport becomes governed by the EDLs, resulting in a system with ion selectivity. This unique feature makes nanochannels ideal for controlling and manipulating ion transport, offering exciting possibilities for cutting-edge technologies and scientific advancements in nanoelectronics and nanosensing^25–28^.

Bipolar nanochannels, drawing inspiration from biological ion nanochannels, have found widespread use in electronic nanochips for ionic current rectification (ICR)^29–33^. Ionic current rectification is the phenomenon in which an electrical current flows more easily in one direction than in the other. This effect can be achieved in nanofluidic devices by creating a heterogeneous surface charge distribution. In one study, Cheng et al.^34^, fabricated nanofluidic diodes using two solid state oxide materials of different isoelectric points, that is, a negatively charged silicon dioxide nanochannel and a positively charged Al_2_O_3_ nanochannel connected in series. The solid oxide nanofluidic diode exhibited high rectification of ion current and achieved record-high rectification factors (ratio of forward current to reverse current) of over 300. The current–voltage property of the device followed the theoretical model quantitatively, except that at low ion concentrations the forward current degraded and the reverse current was greater than theoretical prediction, which can be attributed to access resistance and breakdown of water molecules. The bipolar ionic diode is constructed along the channel wall with two types of surface charges—positive and negative. Under forward bias conditions, ions accumulate at the junction of these surface charges, facilitating the passage of ion current. However, in the reverse bias conditions, the formation of a depletion region at the junction reduces the ion current to nearly zero, resulting in a high ICR value^35–38^. The PNP or NPN ionic nanotransistor is composed of two PN ion diodes interconnected in opposite orientations. These nanotransistors provide a means to control ion transport and modulation in electronic circuits. They offer promising prospects for creating efficient ion-based electronic devices, with potential applications in areas like signal processing, sensing, and energy conversion. The development of these bipolar nanochannels and nanotransistors marks a significant advancement in the field of nanoelectronics and brings us closer to realizing innovative and high-performance nanochip technologies^39–41^.

Daiguji et al.^40^, present a theoretical exploration of ionic transport in nanofluidic channels, emphasizing the development of nanofluidic diodes and transistors. The authors employ the Poisson–Nernst–Planck (PNP) and Navier–Stokes (NS) equations to model ion and fluid flow in a nanochannel with a charged surface. They demonstrate that ionic current modulation is achievable by locally adjusting surface charge density through a gate electrode, laying the foundation for nanofluidic diodes and transistors. In a separate study, Volkov et al.^42^, focus on enhancing spatiotemporal control in life sciences using ion bipolar junction transistors (IBJTs) for constructing delivery circuits. Operating effectively under physiological conditions, IBJTs regulate neurotransmitter delivery in neuronal cells. Despite advancements in integrated circuits, a detailed understanding of IBJT device physics is lacking. The authors address this gap by modeling IBJTs using Poisson’s and Nernst–Planck equations. Their two-dimensional model accurately replicates measurement data characteristics, facilitating the analysis of transistor operation modes and transitions. Successful prediction of the measured threshold voltage enhances the understanding of IBJT operation, forming the basis for discussions on potential miniaturization and device parameter improvements.

Nanotransistors function as miniature switches capable of toggling circuits on and off. This property makes them highly suitable for data storage applications, as they can store vast amounts of information in a compact space. Additionally, nanotransistors are known for their speed, which is crucial in computer systems requiring rapid access to substantial data volumes. They are commonly employed as switches in digital circuits, offering precise control over the flow of electricity due to their quick switching capabilities. Furthermore, nanotransistors find applications as amplifiers. Amplifiers take small electrical signals and magnify their strength. This feature allows nanotransistors to enhance weak signals, enabling efficient signal processing and transmission in various electronic devices and communication systems^43,44^. In summary, nanotransistors, such as the PNP nanotransistor described in this article, have diverse applications ranging from data storage to signal amplification. Their ability to act as fast switches and precise amplifiers makes them indispensable components in modern electronics and communication technologies^45^.

Previous studies by Alinezhad et al.^46^, have explored the behavior of ionic transport in bipolar nanochannels incorporating a soft layer. The findings indicated that at low electrolyte concentrations, there exists an optimal charge value for the soft layer’s cylindrical geometry, which maximizes ICR. This parameter’s value increases at higher electrolyte concentrations with an increase in the charge of the soft layer. In another study by Alinezhad et al.^47^, the effect of electrolyte concentration on ion transport behavior in nanochannels similar to PNP nanotransistors was explored. The results demonstrated the existence of two distinct zones of ion accumulation and depletion at the junction of charged surfaces in PNP nanochannels. By varying the bulk concentration ratio of the electrolyte in the reservoirs, the passing ion current can be increased. These research findings contribute to a deeper understanding of the behavior of ion transport through nanochannels and nanotransistors, providing valuable insights into optimizing their performance for various applications in nanoelectronics, sensing, and data storage. The manipulation of charged surfaces, geometrical parameters, and electrolyte concentrations offers opportunities to tailor and enhance the functionalities of these nanoscale devices.

Among the studies investigating the impact of surface charge density on ion transport behavior in ionic nanotransistors, Singh et al.’s work in 2017 stands out. In this research, they explored the combined influence of surface charge density and nanochannel diameter on the characteristics of the current–voltage (I–V) curve of a nanochannel^48^. The nanochannel featured a positive surface charge on its wall and was equipped with a gate electrode, creating a fluidic field-effect transistor. The results of the study revealed that the ionic current exhibited a linear increase versus the gate potential for narrow nanochannels with high surface charge density. Narrow nanochannels were found to be more effective in controlling the ion current under conditions of high surface charge density. This research sheds light on the intricate interplay between surface charge density and nanochannel diameter in regulating ion transport behavior through ionic nanotransistors. Understanding such interactions is crucial for optimizing the performance of these devices and advancing their applications in diverse fields, including nanoelectronics, biosensors, and lab-on-a-disc technologies^49^.

In 2019, Ferting et al. conducted a study investigating the influence of pH on ionic current in two PNP nanotransistors with different geometries: cylindrical and double-cone^50^. They employed Monte Carlo and Nernst–Planck models to simulate the behavior of these nanotransistors. They found the pH of the electrolyte, which affects the surface charge pattern, a controllable parameter in their experiments. In a neutral environment, there was a depletion of both ionic species, resulting in the lowest passage of ionic current. However, in acidic or alkaline environments, only one of the ion species experienced depletion, leading to the passage of an ionic current. This behavior allowed the PNP nanotransistors to be switched on and/or off by adjusting the pH. The study highlighted the tunability of PNP nanotransistors based on the pH of the electrolyte. By altering the surface charge pattern through pH control, it is possible to modulate the ionic current and effectively turn the nanotransistors on or off. These findings provide valuable insights into the potential applications of PNP nanotransistors as pH-sensitive devices for various purposes, such as biosensing and lab-on-a-chip technologies.

In this research, the impact of surface charge density on ion transport behavior in PNP nanochannels with cylindrical geometry, akin to PNP nanotransistors, has been thoroughly explored. The study focused on key parameters such as ionic concentration, fluid velocity, potential, and ionic current. To achieve this, the current researchers employed a numerical calculation approach based on the finite element method (FEM), which allowed them to solve the steady-state Poisson–Nernst–Planck and Navier–Stokes equations. By employing this numerical method, we were able to gain valuable insights into the behavior of ions within the nanochannels and how the surface charge density affects various ion transport properties^51^. The results obtained from this study contribute to a better understanding of the underlying mechanisms governing ion transport in PNP nanochannels and nanotransistors.

## Physical model

**Figure 1:**
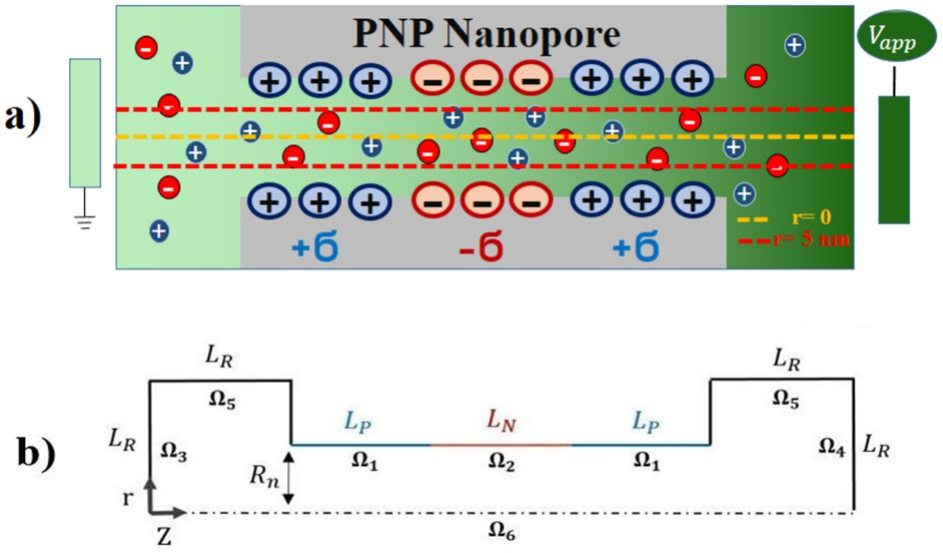
(**a**) Illustration of a PNP nanotransistor with internal surface charge only; (**b**) Schematic of a cylindrical-shaped nanochannel along with applied boundary conditions for simulation. (Note: The red and yellow dashed lines represent cut lines at r = 0 and r = 5 nm respectively).

Figure 1a depicts the nanochannel with a PNP surface charge pattern, which was the focus of this study. In this research, the impact of surface charge density on ionic transfer behavior and electroosmotic flow (EOF) within a PNP cylindrical nanochannel was investigated using the finite element numerical approach. We conducted simulations, obtaining data on the nanochannel conductivity by integrating the total amount of ionic current. The acquired data was then utilized to determine the nanochannel conductivity. For those interested in conducting similar computations, the study provides the necessary information and methodology to apply the finite element approach in examining the influence of surface charge density on ion transport behavior in PNP cylindrical nanochannels.

To find a solution for the system, several assumptions are made. The system is assumed to be steady-state, and the creeping flow regime is considered. The fluid is supposed to be Newtonian, with a viscosity denoted as ${\upmu }_{\text{E}}$. The diffusion coefficients of cations and anions are represented by ${\text{D}}_{\text{j}}$ (where j = 1 for cations $\left({\text{K}}^{+}\right)$ and j = 2 for anions $\left({\text{Cl}}^{-}\right)$), while the electrolyte permittivity is denoted as ${\upvarepsilon }_{\text{E}}$. The hydrodynamic pressure, fluid velocity, electrical potential, and flux of ionic species are represented by $p$, $\mathbf{u}$, $\upphi ,$ and $\mathbf{N}$, respectively. The system’s behavior is indeed formulated using the Poisson–Nernst–Planck equation, which accounts for ion transport in the presence of an electric field, and the corrected Navier–Stokes equations, governing fluid flow in nanofluidic systems. In our study, we specifically investigate the impact of electroosmotic flow (EOF), a phenomenon often overlooked in prior research. This is achieved through the solution of a comprehensive set of coupled equations, including the Poisson, Nernst–Planck, and Navier–Stokes equations. The literature indicates that EOF becomes particularly significant under conditions of medium to high bulk ionic concentration and high electric potential bias. Lin et al.^52^, have demonstrated that neglecting EOF can result in a considerable deviation in rectification ratio, potentially reaching up to 100%. Therefore, in our simulations, we aim to enhance modeling accuracy and better approximate real-world conditions by incorporating this full set of coupled equations. The equations can be expressed as follows^53^:

1 $${\upvarepsilon }_{\text{E}}{\nabla }^{2}\upphi = -{\uprho }_{\text{E}}$$

2 $$\nabla { }.{ }{\mathbf{N}}_{{\mathbf{j}}} = { }\nabla { }.{ }\left( {{\text{c}}_{{\text{j}}} {\mathbf{u}} - {\text{ D}}_{{\text{j}}} \nabla {\text{c}}_{{\text{j}}} - {\text{ D}}_{{\text{j}}} \frac{{{\text{z}}_{{\text{j}}} {\text{Fc}}_{{\text{j}}} }}{{{\text{RT}}}}\nabla \phi } \right) = 0$$

3 $$\nabla .\mathbf{u}=0$$

4 $${\upmu }_{\text{E}}{\nabla }^{2}\mathbf{u}-\nabla \text{p}-{\uprho }_{\text{E}}\nabla\upphi =0$$

The ionic density of the electrolyte, denoted as ${\uprho }_{\text{E}}$, is defined as the sum of ionic species present in the KCl electrolyte. Mathematically, it can be expressed as ${\uprho }_{{\text{E}}} { = }\sum\limits_{{\text{j = 1}}}^{2} {{\text{z}}_{{\text{j}}} {\text{Fc}}_{{\text{j}}} }$, where ${\text{z}}_{\text{j}}$ represents the charge of the ionic species (j = 1 for cations, j = 2 for anions), F is the Faraday constant, and ${\text{c}}_{\text{j}}$ is the concentration of the ionic species in the electrolyte. The nanochannel is connected to two large reservoirs that contain a KCl electrolyte with equal concentrations, as depicted in Fig. 1a. When a voltage is applied from the right electrode, ions migrate into the nanochannel according to the applied voltage signal. According to Fig. 1b, the positive and negative charged lengths of the nanotransistor are denoted as ${\text{L}}_{\text{P }}$ and ${\text{L}}_{\text{N}}$, respectively, while both the radius and the size of the tanks are equal and represented by ${\text{L}}_{\text{R}}$. The radius of the nanochannel is ${\text{R}}_{\text{n}}$. Due to its axial symmetry, the cylindrical nanochannel can be modeled in two dimensions. During the simulation, the model is rotated relative to the axis of symmetry to obtain findings applicable to three-dimensional space. The parameter values utilized in Table 1 are thoroughly justified, ensuring the reliability and accuracy of the simulation results.

**Table 1:** The simulation’s input parameters.

Parameter	Description	Value
${\text{c}}_{0}$	Bulk concentration	$1-100\text{ mM}$
${\upsigma }_{\text{P}}$	Positive surface charge	$1, 5, 20\text{ mC}.{\text{m}}^{-2}$
${\upsigma }_{\text{N}}$	Negative surface charge	$-1,-5,-20\text{ mC}.{\text{m}}^{-2}$
${\text{V}}_{\text{app}}$	Applied voltage	$-1,-\text{0.5,0},\text{0.5,1 V}$
${\text{D}}_{{\text{K}}^{+}}$	Diffusivity of K^+^	$1.96\text{e}-9 {\text{m}}^{2}/\text{s}$
${\text{D}}_{{\text{Cl}}^{-}}$	Diffusivity of Cl^−^	$2.03\text{e}-9 {\text{m}}^{2}/\text{s}$
$\text{e}$	Elementary charge	$1.6022\text{e}-19\text{ C}$
$\text{F}$	Faraday constant	$96500\text{ C}/\text{mol}$
${\text{k}}_{\text{B}}$	Boltzmann constant	$1.381\text{e}-23\text{ J}/\text{K}$
${\text{L}}_{\text{P}}$	Positive surface length	$100\text{ nm}$
${\text{L}}_{\text{N}}$	Negative surface length	$100\text{ nm}$
${\text{L}}_{\text{R}}$	Length and radius of cylindrical reservoirs	$50\text{ nm}$
$\text{R}$	Gas constant	$8.3145\text{ J}/(\text{mol}.\text{K})$
${\text{R}}_{\text{n}}$	Nanochannel radius	$10\text{ nm}$
$\text{T}$	Absolute temperature	$298.15\text{ K}$
${{\upvarepsilon }_{ }}_{\text{E}}$	Relative permittivity of electrolyte	$80$
${{\upvarepsilon }_{ }}_{0}$	Permittivity of vacuum	$8.854\text{e}-12\text{ F}/\text{m}$
$\uprho$	Fluid density	$1000\text{ kg}/{\text{m}}^{3}$
${{\upmu }_{ }}_{\text{E}}$	Fluid viscosity	$0.001\text{ Pa}.\text{s}$
$\text{z}$	Valance of surface charge	$-1, 1$

## Numerical solution

Due to the highly non-linear nature of the partial differential equations involved, an analytical solution to these equations was not feasible. Therefore, numerical methods were employed to solve the equations. To solve Eqs. (1), (2), (3), (4), we need to apply the following boundary conditions:

1. The wall of the nanochannel (${\Omega }_{1},{\Omega }_{2}$) is considered a hard wall with a non-slip condition $\left(\mathbf{u}=0\right)$^54^. Moreover, no ion transfer to the outside is allowed, leading to the condition $\text{n}.{\mathbf{N}}_{\text{j}}=0$^55^. The surface charge on ${\Omega }_{1}$ is ${\upsigma }_{\text{P}}$, and on ${\Omega }_{2}$, it is ${\upsigma }_{\text{N}}$^56^.
2. The concentration in the two tanks is equal to each other and is initialized to the value ${c}_{0}$^57,58^.
3. The ionic concentration at the ends of the reservoirs equals the bulk concentration, which is denoted as ${\text{c}}_{\text{j}}={\text{C}}_{0}$^58^. Additionally, an electrical potential ${\text{V}}_{\text{app}}$ is applied to the bottom wall of the right reservoir $\left({\Omega }_{4}\right)$, while the top wall of the left reservoir $\left({\Omega }_{3}\right)$ is grounded^59^.
4. There is no pressure gradient applied to the system^60^.
5. The side walls of the tanks (${\Omega }_{5}$) are subject to a slip condition $(n.\mathbf{u}=0)$, and there is no ion seepage or charge transfer from them. It is implicitly assumed that there are no viscous effects at the slip wall, and hence, no boundary layer develops. From a modeling point of view, this can be a reasonable approximation if the main effect of the wall is to prevent fluid from leaving the domain^61^.
6. In order to simplify the model, variations in fluid viscosity with surface charge density have been omitted. However, if the voltage range increases, the impact of the electric field on fluid viscosity, known as the viscoelectric effect, should be considered to enhance the accuracy of the model^62,63^.
7. The system exhibits axial symmetry (${\Omega }_{6}$)^64,65^.

It is worth noting that the simulation model has been simplified by replacing the use of a gate electrode (gate voltage) with a constant surface charge density in the gate area. It is important to observe that the surface charge density magnitudes in the gate area (N-type region) and the original channel wall region (P-type region) are exactly the same. For an accurate representation of the nanofluidic transistor’s function, it is important that the surface charge density in the P-type areas remains constant and does not change with the surface charge density of the gate region (N-type region). The application of a voltage causes modulation of the current by the surface charge of the gate area, which is affected by the voltage supplied to the gate.

By applying these boundary conditions, we can proceed with the numerical solution of the coupled Poisson–Nernst–Planck and Navier–Stokes equations using the finite element method. These conditions ensure that the model appropriately represents the physical behavior of the nanotransistor and the flow of ions under the specified setup.

To investigate the ionic current resulting from this system, the ionic current value can be calculated using Eq. (5) by integrating the flux of all ionic species in the system.

5 $${\text{I = }}\int {{\text{F}}\left( {\sum\limits_{{\text{j = 1}}}^{2} {{\text{z}}_{{\text{j}}} {\text{N}}_{{\text{j}}} } } \right) \cdot {\text{ndS}}}$$

where, S is reservoir cross-sectional area.

**Figure 2:**
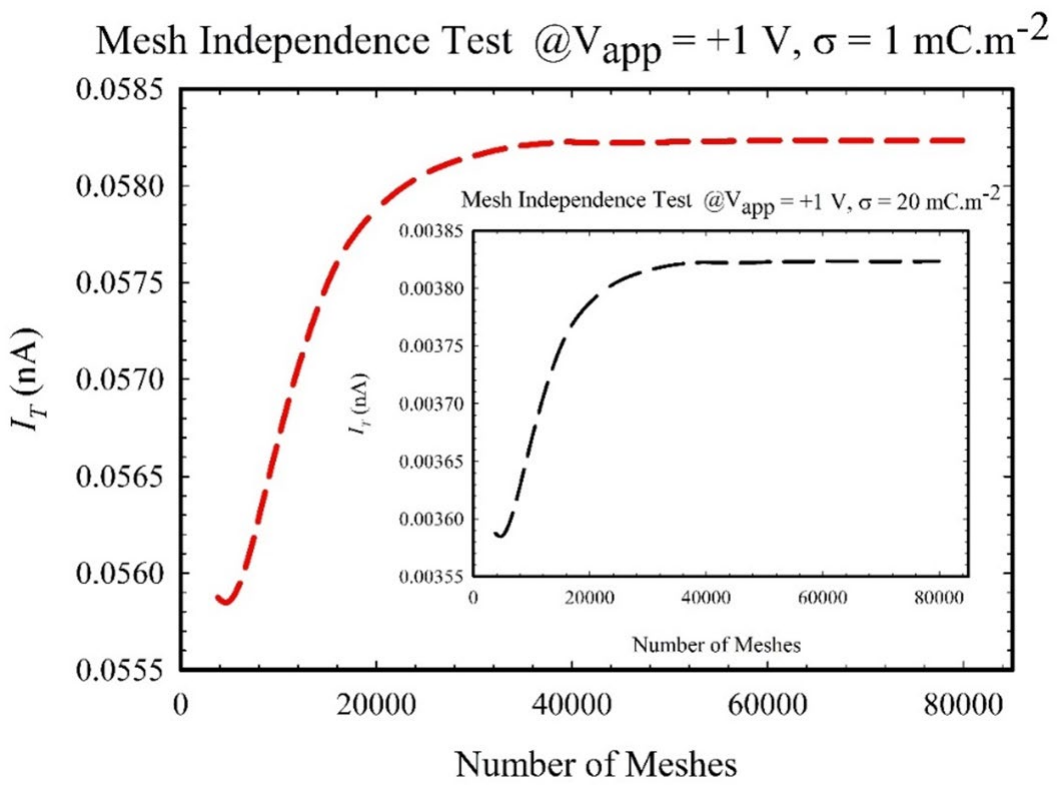
The dependence of the total ionic current magnitude along the nanochannel on the number of mesh elements is illustrated for $\upsigma =1\text{ mC}.{\text{m}}^{-2}$. Additionally, it is noted that the inset highlights the mesh independence test for $=20\text{ mC}.{\text{m}}^{-2}$.

Given the interdependence and high nonlinearity inherent in Eqs. (1), (2), (3), (4), employing suitable numerical methods becomes imperative for their solution. In this study, we utilized the Comsol Multiphysics software (version 6.1a) and employed the high-performance finite element method. The simulation integrated physics modules for electrostatics, transport of diluted species, and creeping flow interfaces, utilizing a combination of triangular and square meshes. To ascertain the optimal mesh count, a mesh-independence study was conducted on the cylindrical geometry. A mesh exceeding 42,000 grids was chosen to ensure precision in the simulation results, as demonstrated in Fig. 2. The mesh-independent study revealed a discrepancy below 0.1% compared to results obtained using 80,000 mesh elements. Specifically, refined meshing was concentrated around the charged wall to accurately capture phenomena associated with electric double layers (EDLs). Moreover, recognizing the significance of the computational domain in theoretical studies, we carefully considered the nanochannel’s length (300 nm) and diameter (20 nm) in this investigation. To mitigate end effects and ensure the reliability and convergence of our results, it was determined that the reservoirs should have a size nearly double that of the nanochannel radius. To verify the adequacy of the reservoir size in preventing far-field ion depletion, we conducted additional simulations with varying reservoir sizes. Table 2 presents the total ionic current in the cylindrical nanochannel under different reservoir sizes. In our simulations, where the reservoir’s radius (length) is 50 nm, we observed a negligible variation of only 0.068% compared to when the reservoir size is 150 nm. This finding indicates that our results are effectively independent of the reservoir edges effect. It is essential to note that, in line with existing literature, for negligible impact on results due to reservoir size, a reservoir size three times the radius of the nanochannel is recommended^66^. Our study adheres to this criterion, ensuring the robustness and reliability of our theoretical outcomes.

**Table 2:** The total ionic current at different reservoir sizes.

Reservoir sizes (nm)	${\text{I}}_{\text{T}}\left(\text{nA}\right)$	Variation (%)
10	$0.05959$	$2.494$
20	$0.05931$	$2.012$
30	$0.05926$	$1.926$
50	$0.05818$	$0.068$
100	$0.05816$	$0.034$
150	$0.05814$	$0.000$

$$\left({\text{V}}_{\text{app}}= +1\text{ V},\upsigma =1 \text{and} 20\text{ mC}.{\text{m}}^{-2}\right)$$.

## Results and discussions

The impact of surface charge density on the axial concentration profiles, velocity, and electric potential within nanochannels incorporating a surface charge arrangement similar to PNP nanotransistors has been studied. The investigation was conducted at two distinct locations within the nanochannel: the center ($\text{r}=0$) and a distance of $\text{r}=5\text{ nm}$ from the center. The study was carried out under two applied voltages of ${\text{V}}_{\text{app}}=\pm 1\text{ V}$. In a separate part of the research, the influence of surface charge density on the I–V characteristics and the ionic current of both cations and anions was examined under the same applied voltages of ${\text{V}}_{\text{app}}=\pm 1\text{ V}$. The I–V graph represents the relationship between the applied voltage and the ionic current passing through the nanochannel. Analyzing the I–V curves for different surface charge densities provided insights into the ion current behavior and rectification phenomena. This research contributes to a better understanding of how surface charge density affects ion transport behavior in nanochannels with PNP-like surface charge arrangements.

**Figure 3:**
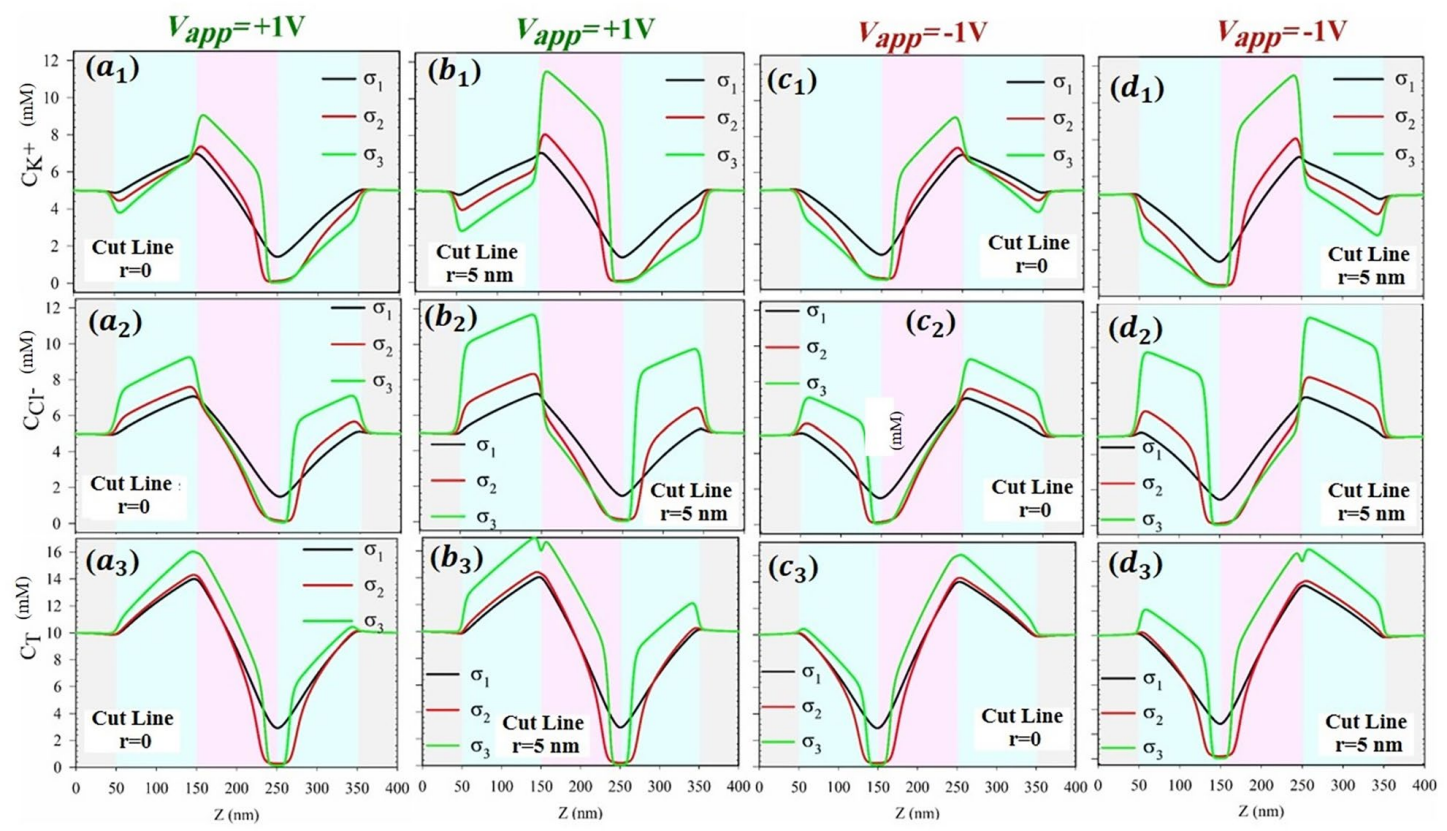
Axial distribution of concentration of for (${\text{a}}_{1},{\text{b}}_{1},{\text{c}}_{1},{\text{d}}_{1}$) cations, (${\text{a}}_{1},{\text{c}}_{1}$) at cut line $\text{r}=0$, (${{\text{b}}_{1},\text{d}}_{1}$) at cut line $\text{r}=5\text{ nm}$; (${\text{a}}_{2},{\text{b}}_{2},{\text{c}}_{2},{\text{d}}_{2}$) anions, (${\text{a}}_{2},{\text{c}}_{2}$) at cut line $\text{r}=0$, (${{\text{b}}_{2},\text{d}}_{2}$) at cut line $\text{r}=5\text{ nm}$; and (${\text{a}}_{3},{\text{b}}_{3},{\text{c}}_{3},{\text{d}}_{3}$) total ions (${\text{C}}_{\text{T}} \left(\text{mM}\right)$), (${\text{a}}_{3},{\text{c}}_{3}$) at cut line $\text{r}=0$, (${{\text{b}}_{3},\text{d}}_{3}$) at cut line $\text{r}=5\text{ nm}$ at different surface charge densities (${\upsigma }_{1}=1\text{ mC}.{\text{m}}^{-2}$ black line, ${\upsigma }_{2}=5\text{ mC}.{\text{m}}^{-2}$ red line and ${\upsigma }_{3}=20\text{ mC}.{\text{m}}^{-2}$ green line) at ${\text{V}}_{\text{app}}=\pm 1\text{ V}$. (It should be mentioned that, in all panels, the bulk concentration is 5 mM).

In the initial stage of the research, the concentration distribution of cations and anions, as well as the total ions, was examined along the length of the nanochannel at cut lines of $\text{r}=0$ and $\text{r}=5\text{ nm}$ (as shown in Fig. 3). It should be mentioned that ${\text{C}}_{\text{T}}$ represents the total concentration, which includes both the bulk concentration for cations and anions. Under the applied voltage of ${\text{V}}_{\text{app}}=+1\text{ V}$, locations of ion accumulation and depletion occurred at the junctions of surface charges, specifically at $\text{Z}=150\text{ nm}$ and $\text{Z}=250\text{ nm}$, respectively. By changing the applied potential from ${\text{V}}_{\text{app}}=+1\text{ V}$ to ${\text{V}}_{\text{app}}=-1\text{ V}$ at the active electrode, the positions of ion accumulation and depletion points were reversed to $\text{Z}=250\text{ nm}$ and $\text{Z}=150\text{ nm}$, respectively (as previously explained in detail^47^). The objective of this part was to investigate the influence of surface charge density on the ion concentration distribution along the length of the nanochannel at cut lines of $\text{r}=0$ and $\text{r}=5\text{ nm}$. The results revealed that the concentration of cations (${\text{a}}_{1},{\text{b}}_{1},{\text{c}}_{1},{\text{d}}_{1}$) reached its maximum in proximity to the negatively charged surface, exhibiting an increase with the rise in surface charge density. Conversely, the concentration of anions (${\text{a}}_{2},{\text{b}}_{2},{\text{c}}_{2},{\text{d}}_{2}$) reached its peak near the positively charged surface, demonstrating an augmentation with the increase in surface charge density. This observation is consistent with the expected opposite trends for cations and anions due to their opposite polarity. Moreover, in various states, both the accumulation and depth of ion depletion increased with surface charge density. By comparing the shapes of (${\text{a}}_{1},{\text{a}}_{2},{\text{c}}_{1},{\text{c}}_{2}$) with $({\text{b}}_{1},{\text{b}}_{2},{\text{d}}_{1},{\text{d}}_{2}$), it was concluded that the concentration of ions increased as they approached the surface with opposite charge. Furthermore, at the cut line of $\text{r}=5\text{ nm}$, it was observed that a higher concentration of ions entered the nanochannel with increasing surface charge density (as represented by ${\text{a}}_{3},{\text{b}}_{3},{\text{c}}_{3},{\text{d}}_{3}$).

**Figure 4:**
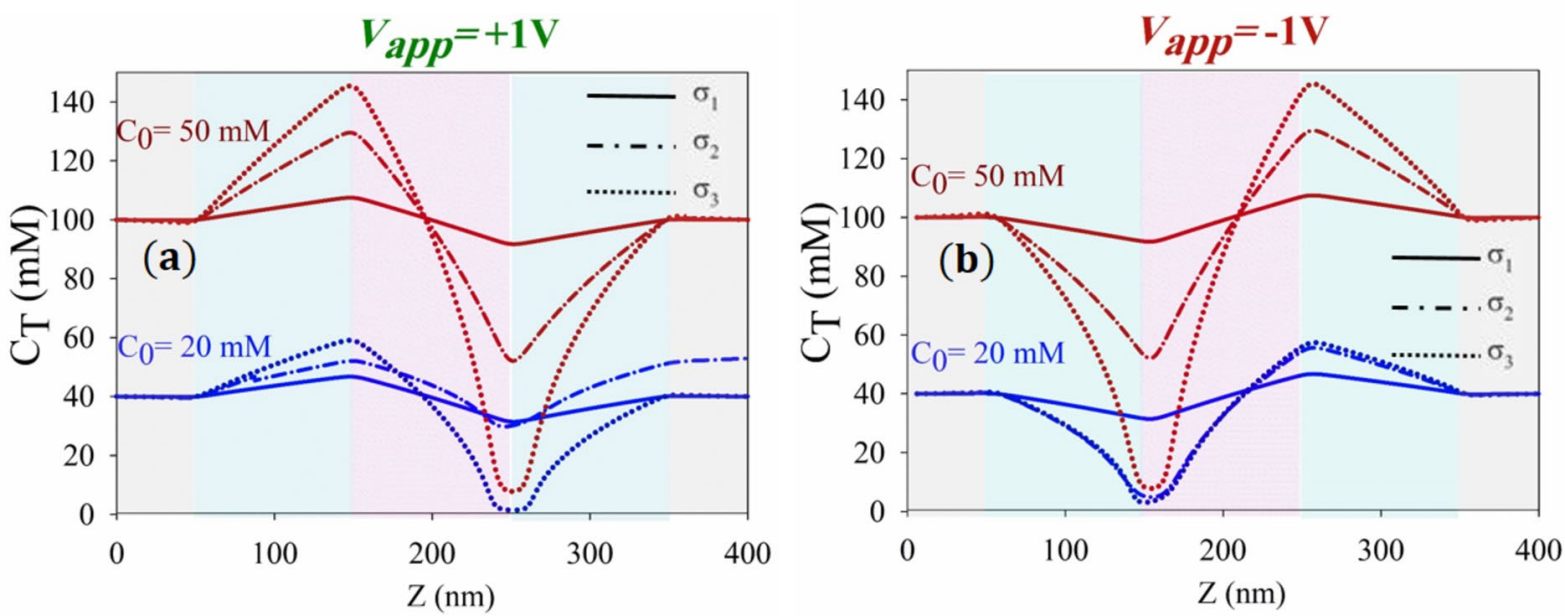
Axial distribution of total concentration of ions (${\text{C}}_{\text{T}}$) for (**a**) ${\text{V}}_{\text{app}}=+1\text{ V}$ and (**b**) ${\text{V}}_{\text{app}}=-1\text{ V}$ at different surface charge densities (${\upsigma }_{1}=1\text{ mC}.{\text{m}}^{-2}$,${\upsigma }_{2}=5\text{ mC}.{\text{m}}^{-2}$ and ${\upsigma }_{3}=20\text{ mC}.{\text{m}}^{-2}$).

From an alternative perspective, Fig. 4 delves into the distribution of ionic concentration under bulk concentrations exceeding $5\text{ mM}$ (${\text{C}}_{0}=20\text{ mM and }50\text{ mM}$), considering various values of surface charge density, and subject to two distinct applied voltages (${\text{V}}_{\text{app}}=\pm 1\text{ V}$). These results diverge from those depicted in the preceding figure. As bulk concentration rises, a notable trend emerges: under higher surface charge densities (${\upsigma }_{3}$), the depth of ion depletion increases, contrasting with the behavior observed under ${\upsigma }_{2}$ in Fig. 3. Notably, at elevated concentrations, the influence of surface charge on ion absorption diminishes, primarily impacting the depth of ion depletion. This observation underscores the intricate interplay between bulk concentration, surface charge density, and ion behavior within the nanochannel environment.

From another point of view, when the bulk ion concentration and surface charge are increased, the electric double layer (EDL) near the nanochannel surface becomes more pronounced. This results in a stronger electric field within the EDL, which in turn enhances the ion depletion effect. Specifically, the increased surface charge attracts counter-ions (opposite charge) and repels co-ions (same charge), leading to a significant reduction in the concentration of co-ions near the surface. This ion depletion is further influenced by the principle of electroneutrality. As one type of ion is depleted, the system compensates by depleting other ionic species to maintain overall charge neutrality. This is analogous to the ion concentration polarization (ICP) phenomenon, where the selective transport of ions through a membrane or nanostructure leads to the formation of distinct depletion and enrichment regions.

In summary, the combined effect of higher bulk ion concentration and increased surface charge intensifies the electric field within the EDL, thereby enhancing the depletion of specific ions. This depletion is balanced by the corresponding depletion of other ionic species to preserve electroneutrality, resulting in a more pronounced depletion region.

**Figure 5:**
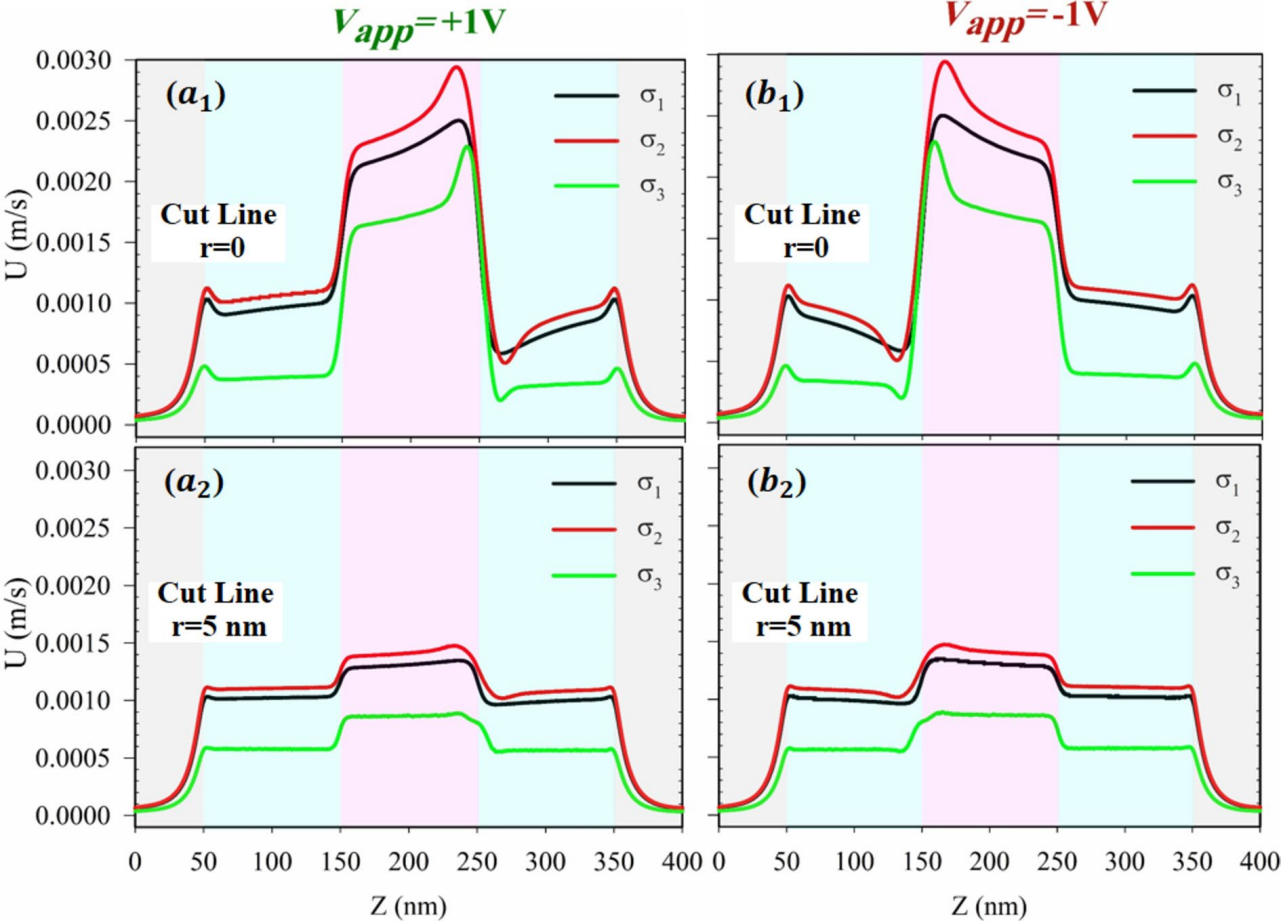
The axial velocity distribution for various surface charge densities (${\upsigma }_{1}=1\text{ mC}.{\text{m}}^{-2}$ black line,${\upsigma }_{2}=5\text{ mC}.{\text{m}}^{-2}$ red line and ${\upsigma }_{3}=20\text{ mC}.{\text{m}}^{-2}$ green line) for (${\text{a}}_{1},{\text{b}}_{1}$) at cut line $\text{r}=0$ and (${\text{a}}_{2},{\text{b}}_{2}$) at cut line $\text{r}=5\text{ nm}$ at ${\text{V}}_{\text{app}}=\pm 1\text{ V}$. (It should be mentioned that, in all panels, the bulk concentration is 5 mM).

Figure 5 illustrates the impact of surface charge density on the axial velocity of ions $\left(\text{U} [m/s]\right)$ in the PNP nanochannel at cut lines $\text{r}=0$ and $\text{r}=5\text{ nm}$. The results show that a higher velocity is observed in the range of $\text{Z}=150\text{ nm}$ to $\text{Z}=250\text{ nm}$ (near the negative surface charge) compared to other locations along the nanochannel's length. At the locations where ions experience depletion (at $\text{Z}=250\text{ nm}$ under ${\text{V}}_{\text{app}}=+1\text{ V}$ and at $\text{Z}=150\text{ nm}$ under ${\text{V}}_{\text{app}}=-1\text{ V}$), the velocity reaches its maximum value. The effect of surface charge density on the velocity is evident in Fig. 5, where the medium surface charge density (${\upsigma }_{2}=5\text{ mC}.{\text{m}}^{-2}$) leads to higher velocity along the nanochannel compared to other surface charge density values. As the surface charge density increases beyond this medium value, the velocity along the nanochannel gradually decreases. Comparing the velocity profiles at cut lines of $\text{r}=0$ and $\text{r}=5\text{ nm}$ reveals that as the nanochannel’s surface is approached, fluctuations in the velocity reach their lowest values. The velocity of ions decreases near the surface of the nanochannel, and this reduction in velocity is more pronounced in the middle surface with a negative charge ($\text{Z}=150\text{ nm}$ to $\text{Z}=250\text{ nm}$) of the nanochannel (as observed in the panels ${\text{a}}_{2}$ and ${\text{b}}_{2}$ of Fig. 5).

From another point of view, as surface charge density increases beyond an medium value, electrostatic repulsion between similarly charged ions within the electric double layer near the charged surface intensifies, leading to a thicker double layer and stronger repulsive forces. This impedes ion movement through the nanochannel, resulting in a gradual decrease in velocity. Additionally, higher surface charge densities can enhance ion adsorption onto the charged surface and alter the distribution of counterions within the electric double layer. The manipulation of surface charge density has the potential to exert influence on the electroosmotic flow dynamics within a nanochannel. As the surface charge density experiences an increase, it can instigate a heightened level of interaction between the fluid and the channel walls, consequently resulting in a reduction of the overall fluid velocity. This phenomenon can be attributed to the alterations in the electrokinetic effects and ionic arrangements within the nanochannel, leading to significant changes in the flow behavior. It is important to note that while the viscoelectric effect is not explicitly included in our current model, it could become significant at higher surface charge densities, suggesting a potential area for future work to enhance model accuracy.

On the other hand, the level of fluid velocity is intricately tied to the specific application of the nanochannel system. For instance, in the context of lab-on-a-chip devices utilized for fluid transport in medical diagnostics, the challenge of fluid pumping often arises, presenting an inherent lack of control^67^. However, through the implementation of surface charge control, it becomes feasible to regulate the fluid velocity, thereby enhancing the adaptability of the fluid pumping method in sensitive applications. Consequently, the focal point shifts from simply attaining higher fluid velocity to the crucial aspect of controlling the velocity, which holds paramount significance.

**Figure 6:**
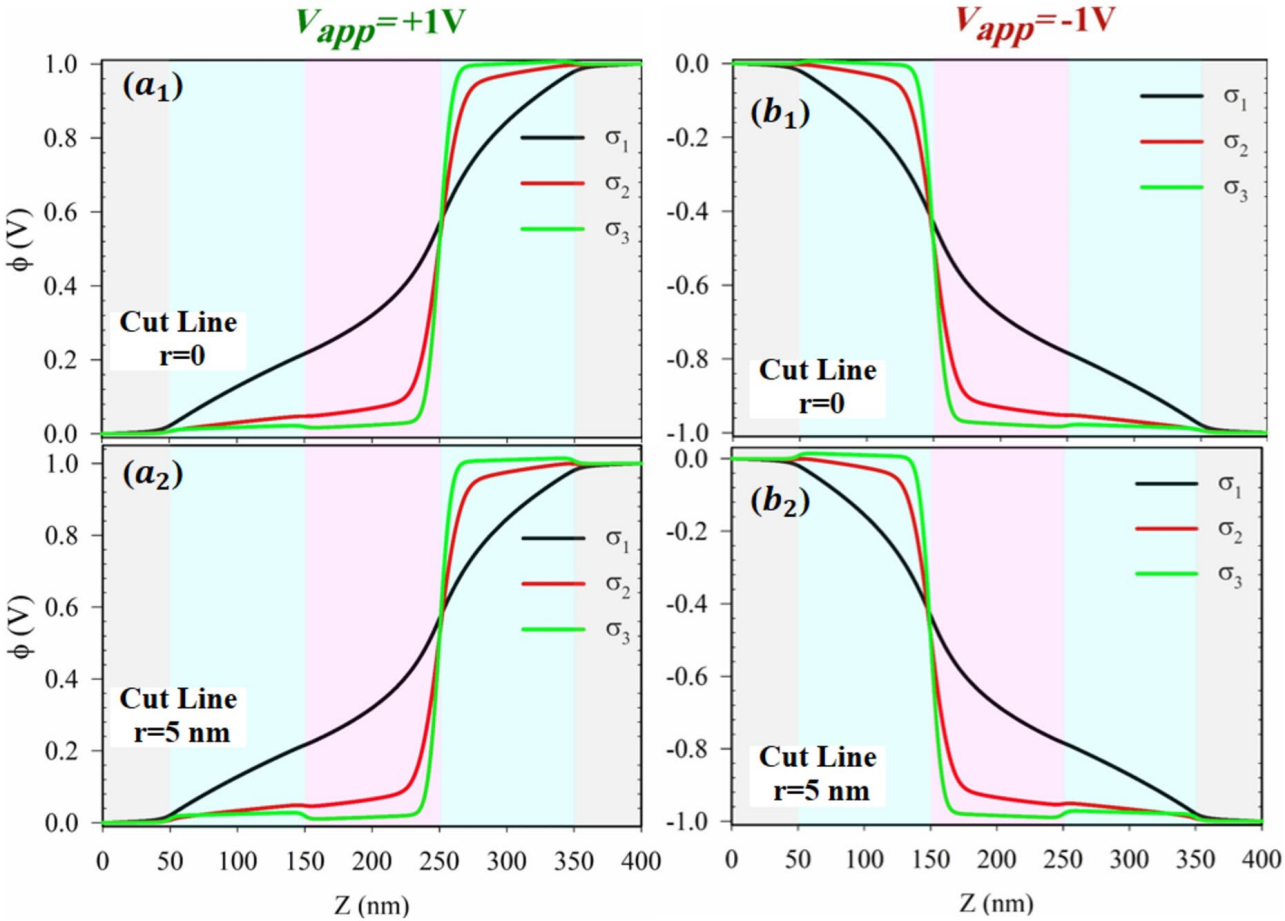
The axial electrical potential distribution versus surface charge densities $({\upsigma }_{1}=1\text{ mC}.{\text{m}}^{-2}$ black line,${\upsigma }_{2}=5\text{ mC}.{\text{m}}^{-2}$ red line and ${\upsigma }_{3}=20\text{ mC}.{\text{m}}^{-2}$ green line) on for (${\text{a}}_{1},{\text{b}}_{1}$) at cut line $\text{r}=0$ and (${\text{a}}_{2},{\text{b}}_{2}$) at cut line $\text{r}=5\text{ nm}$ at ${\text{V}}_{\text{app}}=\pm 1\text{ V}$. (It should be mentioned that, in all panels, the bulk concentration is $5\text{ mM}$).

Figure 6 presents the electrical potential profile within the nanochannel along its length at different surface charge density values under two applied voltages of ${\text{V}}_{\text{app}}=\pm 1\text{ V}$. At ${\text{V}}_{\text{app}}=+1\text{ V}$, the significant changes in potential occur at $\text{Z}=250\text{ nm}$, whereas at ${\text{V}}_{\text{app}}=-1\text{ V}$, these changes occur at $\text{Z}=150\text{ nm}$. These points correspond to locations where ions are depleted within the nanochannel. At these depletion regions, the length of the EDL increases, and the effect of surface charge density on the potential inside the nanochannel becomes more prominent. Subsequently, the electrical potential changes are primarily controlled by the charged surface. Furthermore, with an increase in the surface charge density, the changes in electrical potential at the ion depletion locations become more pronounced. Conversely, at lower surface charge densities, the electrical potential changes almost uniformly between the two electrodes. As the surface charge density increases, its influence on the electrical potential profile inside the nanochannel becomes more significant than the external potential applied by the electrode. Upon comparing (${\text{a}}_{1},{\text{b}}_{1}$) with $({\text{a}}_{2},{\text{b}}_{2}$) in Fig. 6, it is evident that the electrical potential profiles at different surface charge density values in cut lines of $\text{r}=0$ and $\text{r}=5\text{ nm}$ do not exhibit substantial differences.

**Figure 7:**
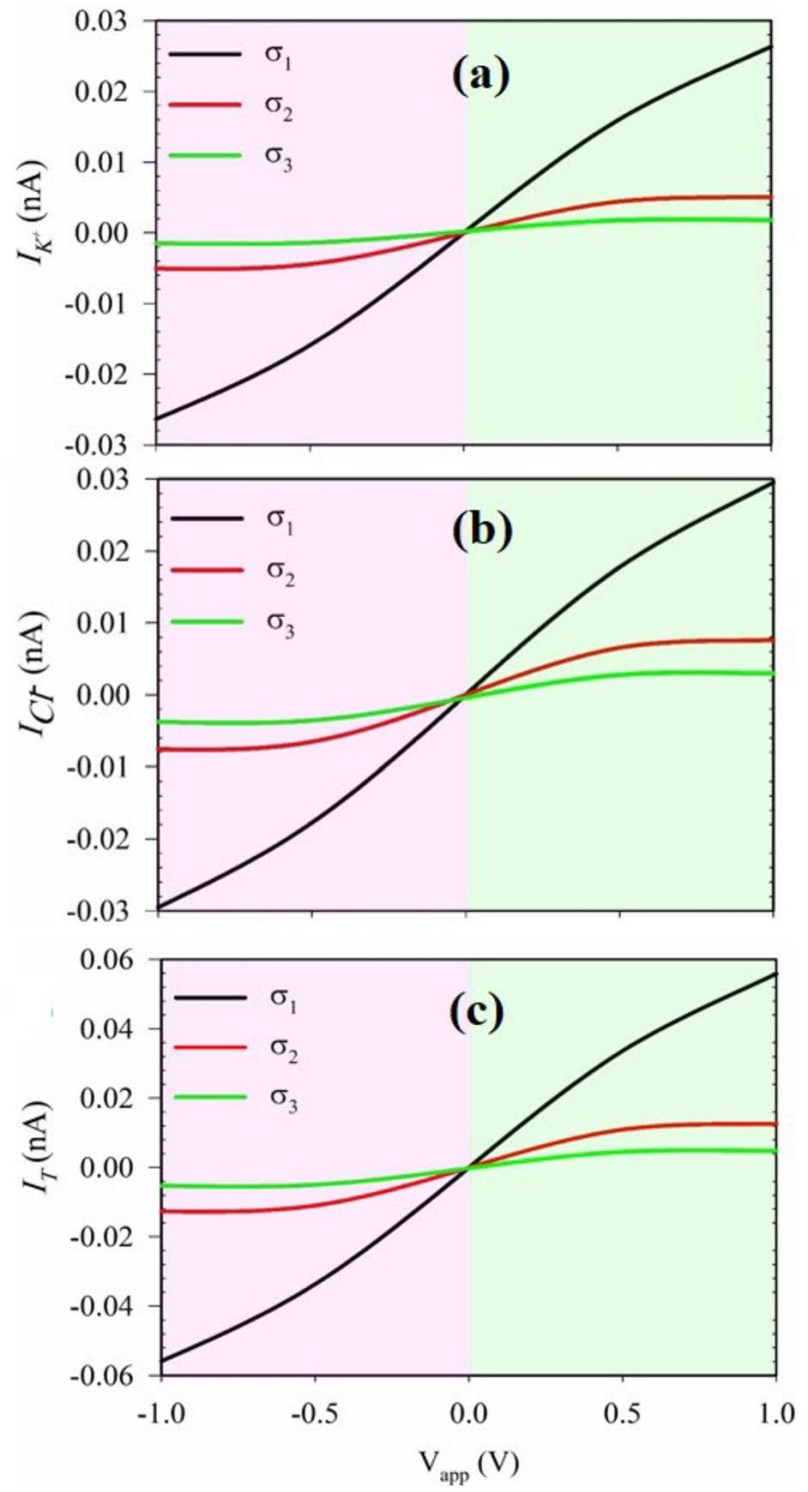
I–V curves for various surface charge densities $({\upsigma }_{1}=1\text{ mC}.{\text{m}}^{-2}$ black line,${\upsigma }_{2}=5\text{ mC}.{\text{m}}^{-2}$ red line and ${\upsigma }_{3}=20\text{ mC}.{\text{m}}^{-2}$ green line) in PNP cylindrical nanochannels for )**a**) cations, (**b**) anions and (**c**) total ions. (It should be mentioned that, in all panels, the bulk concentration is 5 mM).

Figure 7 presents the passing ionic currents for ${\text{Cl}}^{-}$ and ${\text{K}}^{+}$ ions, along with the total ionic current, across a range of applied voltages from $+1\text{ V}$ to $-1\text{ V}$, and at different surface charge density values. The results demonstrate that as the surface charge density increases, the levels of passing ionic currents decrease, particularly at high voltage values. This phenomenon allows for the modulation of the ion current between an off-state and an on-state by varying the surface charge density in PNP nanotransistors. At high surface charge density, the system operates in the off-state, while at low levels of surface charge density, it enters an on-state. Moreover, the presence of a higher positive charge on the surface of this nanochannel leads to an increased ionic current of negatively charged ions into the nanochannel. This interplay between surface charge density and ion current has significant implications for controlling the ion transport behavior in PNP nanotransistors. The ability to switch the nanotransistor between off and on states through changes in surface charge density is crucial for various applications, such as data storage and signal processing. Overall, these findings provide valuable insights into how surface charge density influences the ion current behavior and ion selectivity in PNP nanotransistors.

Additionally, Increasing the surface charge of a PNP nanotransistor results in a reduction of both the total current and each individual current. This is due to the operational principles of PNP transistors, where the majority charge carriers are holes. As the surface charge intensifies, the interaction between the holes and the channel walls increases, leading to an overall decrease in current flow. This behavior aligns with the fundamental characteristics of PNP transistors, where the manipulation of surface charge density intricately controls the current flow. Therefore, the heightened surface charge effectively impedes the current flow, consistent with the operational traits of PNP transistors as majority charge carriers.

**Figure 8:**
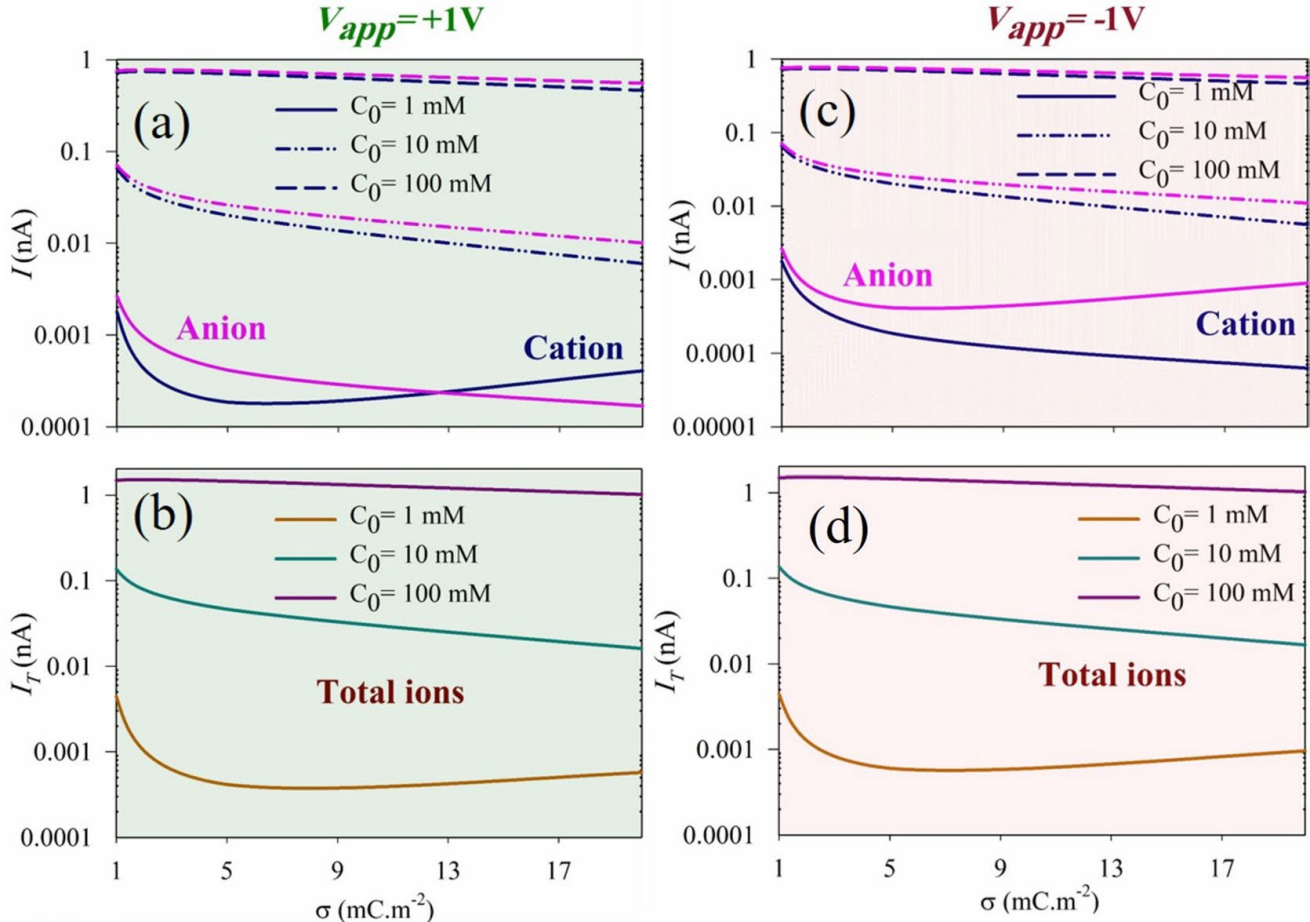
$\text{I}-\upsigma$ curves for various bulk concentrations $({\text{C}}_{0}=1\text{ mM},10\text{ mM and }100\text{ mM}$) in PNP cylindrical nanochannels for )**a**,**c**) cations and anions, (**b**,**d**) total ions, at ${\text{V}}_{\text{app}}=+1\text{ V}$ (**a**,**b**) and ${\text{V}}_{\text{app}}=-1\text{ V}$ (**c**,**d**). (It is important to note that in panels (**a**,**b**), the solid line, the dashed line, and the dashed-dotted line represent ${\text{C}}_{0}=1\text{ mM},10\text{ mM and }100\text{ mM}$, respectively).

Figure 8 demonstrates the effect of surface charge density on the currents of cations and anions at three different electrolyte concentrations under applied voltages. Regardless of the polarity of the applied voltage, an increase in electrolyte concentration results in higher ionic species inside the nanochannel, leading to an overall increase in the ionic current. At high concentrations of electrolyte (${\text{C}}_{0}=10, 100\text{ mM}$) where the EDL is weak, a higher surface charge density causes a decrease in the ionic current. However, at low concentrations of electrolyte (${\text{C}}_{0}=1\text{ mM}$) where the EDL is thick, different behavior is observed. Initially, as the surface charge density increases, the ionic current decreases until it reaches a minimum value at an medium surface charge. Subsequently, with a further increase in surface charge density, the passing ionic current starts to increase again. In the case of a PNP surface charge arrangement, more anions than cations enter the nanochannel, resulting in a higher current of anions. The ion depletion area plays a critical role in controlling the ionic current, with a larger discharge area leading to a decrease in ionic current. At higher electrolyte concentrations, an increase in the surface charge creates a larger ion depletion area, leading to reduced ionic current. In contrast, at low electrolyte concentrations, the overlapping of double layers occurs due to the extended length of the EDLs. Initially, at low surface charge density values, an increase in surface charge density leads to the formation of a larger ion depletion zone, thereby reducing the ion current. In this scenario, the effect of the charged surface plays a significant role in creating the depletion zone. However, as the surface charge density increases to higher values, the influence of the charged surface diminishes. Instead, higher concentrations of ions entering the nanochannel result in a smaller depletion zone, consequently increasing the ion current.

## Conclusions

In this study, we investigated the impact of surface charge density on ion transport behavior, encompassing the concentration, fluid velocity, electrical potential, and ionic current, within cylindrical nanochannels employing a PNP charge arrangement. The operational characteristics of the system are defined through the application of the Poisson–Nernst–Planck equations, elucidating ion transport in an electric field, along with the Navier–Stokes (Brinkman) equations, regulating fluid movement within porous materials. Owing to the profoundly non-linear attributes of the partial differential equations at play, deriving an analytical solution for these equations proved impractical. Consequently, numerical methods were utilized to resolve the equations. The pivotal outcomes obtained through this research are outlined below:

1. Our findings indicate a direct correlation between surface charge density and the depth of the ion depletion zone within the system. Higher surface charge density is associated with an increased depth of the ion depletion zone, leading to reduced ionic velocities and a consequent down in the overall ion current passing through the nanochannel.
2. Nonetheless, a unique behavior was noted at extremely low concentrations (${\text{C}}_{0}=1\text{ mM}$). At an optimum surface charge density, the ionic current experiences minimization. Subsequently, with a further increase in surface charge density, a direct relationship is established with the ionic current.
3. The findings revealed a peak in cation concentration near the negative surface charge, exhibiting an upward trend with increased surface charge density. A parallel pattern was observed in anion concentration. Both ion accumulation and the depth of ion depletion intensified with rising surface charge density.
4. Comparative analysis indicated elevated ion concentrations near surfaces with opposite charges. Additionally, at $\text{r}=5\text{ nm}$, heightened surface charge density correlated with an increased influx of ions into the nanochannel.
5. The results highlight a higher velocity within the range of $\text{Z}=150\text{ nm}$ to $\text{Z}=250\text{ nm}$ (proximate to the negative surface charge) compared to other locations along the nanochannel. Maximum velocity occurs at locations of ion depletion.
6. Under ${\text{V}}_{\text{app}}=+1\text{ V}$, substantial potential drop occurs at $\text{Z}=250\text{ nm}$, and under ${\text{V}}_{\text{app}}=-1\text{ V}$, these drops happen at $\text{Z}=150\text{ nm}$—corresponding to ion depletion zones. The EDL length increases at these points, underscoring the impact of surface charge density on the nanochannel potential. As surface charge density increases, potential changes at ion depletion locations become more pronounced. Conversely, lower surface charge densities yield nearly linear potential changes between electrodes.
7. The results indicate that increasing surface charge density decreases ionic currents, especially at high voltages. This allows modulation of ion current between off and on states in PNP nanotransistors. Higher surface charge density induces an off-state, while lower levels induce an on-state. A higher positive charge on the nanochannel surface enhances the influx of negatively charged ions. This interplay has significant implications for controlling ion transport in PNP nanotransistors, essential for applications like data storage and signal processing. Overall, these findings offer valuable insights into the impact of surface charge density on ion current behavior and selectivity in PNP nanotransistors.

These findings offer valuable insights into the complex relationship between surface charge density and ion transport in PNP-arranged nanochannels. The observed variations in ion concentration, fluid velocity, and current have potential implications for optimizing nanochannel-based devices, particularly in applications demanding precise regulation of ionic currents, like nanoelectronics, sensing, and data storage. The ability to control ion transport via surface charge density manipulation holds significance for enhancing the performance and versatility of nanochannel-based devices. Understanding the impact of surface charge density on ion transport allows researchers to tailor nanochannel design for desired functionalities, advancing the fields of nanoelectronics and nanofluidics.

## Data Availability

The data that support the findings of this study are available from the corresponding author upon reasonable request.
